# Using statistical parametric mapping to assess the association of duty factor and step frequency on running kinetic

**DOI:** 10.3389/fphys.2022.1044363

**Published:** 2022-12-05

**Authors:** Aurélien Patoz, Thibault Lussiana, Bastiaan Breine, Eliott Piguet, Jonathan Gyuriga, Cyrille Gindre, Davide Malatesta

**Affiliations:** ^1^ Institute of Sport Sciences, University of Lausanne, Lausanne, Switzerland; ^2^ Research and Development Department, Volodalen Swiss Sport Lab, Aigle, Switzerland; ^3^ Research and Development Department, Volodalen, France; ^4^ Research Unit EA3920 Prognostic Markers and Regulatory Factors of Cardiovascular Diseases and Exercise Performance, Health, Innovation Platform, University of Franche-Comté, Besançon, France; ^5^ Department of Movement and Sports Sciences, Ghent University, Ghent, Belgium

**Keywords:** biomechanics, running pattern, spring-mass model, leg stiffness, ground reaction force

## Abstract

Duty factor (DF) and step frequency (SF) were previously defined as the key running pattern determinants. Hence, this study aimed to investigate the association of DF and SF on 1) the vertical and fore-aft ground reaction force signals using statistical parametric mapping; 2) the force related variables (peaks, loading rates, impulses); and 3) the spring-mass characteristics of the lower limb, assessed by computing the force-length relationship and leg stiffness, for treadmill runs at several endurance running speeds. One hundred and fifteen runners ran at 9, 11, and 13 km/h. Force data (1000 Hz) and whole-body three-dimensional kinematics (200 Hz) were acquired by an instrumented treadmill and optoelectronic system, respectively. Both lower DF and SF led to larger vertical and fore-aft ground reaction force fluctuations, but to a lower extent for SF than for DF. Besides, the linearity of the force-length relationship during the leg compression decreased with increasing DF or with decreasing SF but did not change during the leg decompression. These findings showed that the lower the DF and the higher the SF, the more the runner relies on the optimization of the spring-mass model, whereas the higher the DF and the lower the SF, the more the runner promotes forward propulsion.

## Introduction

The running pattern was defined to be multifactorial and as being the product of overall action of human body as early as 1985 (Subotnick, 1985). Indeed, foot placement, arm swing, body angle, rear leg lift, and stride length were suggested to be considered together (Subotnick, 1985). The running pattern was also described as a global and dynamic system (Gindre et al., 2016). For this reason, the running pattern has been analyzed globally by some researchers. For instance, McMahon et al. (1987) defined running with increased knee flexion and long ground contact time ( $t_{c}$ ) as *Groucho running* while Arendse et al. (2004) defined running with aligned acromion, greater trochanter, and lateral malleolus as well as short $t_{c}$ as *Pose running*. As another example, running with either a midfoot or forefoot strike pattern, short stride length, and with the body slightly leaning forward has been named *Chi running* (Dreyer and Dreyer, 2009).

More recently, the synthetic review of van Oeveren et al. (2021) proposed that the full spectrum of running patterns could be described by combining two temporal variables: step frequency (SF) and duty factor (DF). The DF variable represents the product of $t_{c}$ and stride frequency, where stride frequency is equivalent to approximately half of SF, with less than 4% differences in step times between right and left sides seen in competitive, recreational, and novice runners between 8 and 12 km/h (Mo et al., 2020). Hence, DF reflects the relative contribution of $t_{c}$ to the running stride (Minetti, 1998; Folland et al., 2017). According to van Oeveren et al. (2021), knowing DF and SF allows to categorize running patterns in one of five distinct categories, namely “stick”, “bounce”, “push”, “hop”, and “sit”, but keeping in mind that running patterns operate along a continuum.

The importance of DF and SF in determining running patterns corroborates previous findings. For instance, Beck et al. (2020) showed that DF is functionally representative of global biomechanical behavior, considering the duration of force production (which takes place during $t_{c}$ ) and its cycle frequency (stride frequency). Moreover, DF was used to categorize runners with distinct running patterns (Lussiana et al., 2019; Patoz et al., 2019; Patoz et al., 2020). High and low DF runners were shown to use different running strategies (Lussiana et al., 2019; Patoz et al., 2020). Indeed, low DF runners exhibited a more symmetrical running step, anterior (midfoot and forefoot) strike pattern, and extended lower limb during $t_{c}$ than high DF runners, whereas the latter exhibited greater lower limb flexion during $t_{c}$ , more rearfoot strike pattern, and less vertical oscillation of the whole-body center of mass (COM) to promote forward propulsion than low DF runners (Lussiana et al., 2019; Patoz et al., 2020). Despite these spatiotemporal and kinematic differences, the two DF groups demonstrated similar running economy, indicating the two strategies are energetically equivalent at endurance running speeds (Lussiana et al., 2019). This would suggest that the two DF groups may optimize differently their running pattern, i.e., high DF runners promotes forward propulsion (pulley system) whereas low DF runners optimized the spring-mass model (Lussiana et al., 2019). This statement was further explored by investigating the relationships between DF and force-length relationship and leg stiffness ( $k_{leg}$ ).

In relation to SF, this variable can reveal individual strategies to increase running speed (Dorn et al., 2012) or achieve top-end running speeds (Salo et al., 2011). Indeed, the consistency in SF was shown to decrease as speed differences increased (tested running speeds: 10–18 km/h) (Patoz et al., 2022) and each runner was shown to self-optimize his step length over SF ratio (Hunter et al., 2017; van Oeveren et al., 2021). Even in subgroups of individuals with similar sprint velocities, a range of SF and step length combinations are present (Hunter et al., 2004). In addition, SF was shown to be more variable in novice than expert runners, independently of the running speed (10 and 15 km/h) (Fadillioglu et al., 2022). Furthermore, Bonnaerens et al. (2021) demonstrated that external forces were lower in recreational runners that run with higher DF and SF values (although non-significant for SF).

These previous studies investigated the association of DF or SF on running biomechanics using summary metrics, i.e., specific temporal focus like foot-strike, mid-stance, or toe-off, of signals such as the whole-body COM trajectory or the lower limb angles during $t_{c}$ (Lussiana et al., 2019; Patoz et al., 2020). This reduction to summary-metric space is not strictly necessary because statistical hypothesis testing can also be conducted in a continuous manner (Pataky, 2012). Indeed, one-dimensional biomechanical curves such as the ground reaction force signals are registrable and their fluctuations can be described and, then, compared expressing them as a function of the normalized stance phase duration (Cavanagh and Lafortune, 1980; Sadeghi et al., 2003). In this case, statistical analysis can be conducted on the original registered curves using statistical parametric mapping (SPM) (Friston et al., 2007), which was recently applied to the field of biomechanics (Pataky, 2010). SPM has the advantages to consider the signal as a whole and presents the results directly in the original sampling space. For this reason, the spatiotemporal biomechanical context is immediately apparent, and allows direct visualization of where do significant differences occur during $t_{c}$ (Pataky, 2012).

Therefore, the first purpose of the present study was to investigate the association of DF and SF on the vertical and fore-aft ground reaction force signals for treadmill runs at several endurance running speeds using SPM. The second purpose of this study was to investigate the association of DF and SF on variables derived from the vertical and fore-aft ground reaction force signals, i.e., impact (*F*_*z*,impact_), active (*F*_*z*,max_), braking (*F*_brake,min_), and propulsive (*F*_prop,max_) peaks (Luo et al., 2019). Besides, force related variables that additionally consider the temporal aspect of the running step were also considered because the latter can vary with DF and SF. The third purpose of the present study was to investigate the association of DF and SF on the force-length relationship (Gill et al., 2020) and $k_{leg}$ (Liew et al., 2017).

We hypothesized that 1) a lower DF should be associated to higher vertical and fore-aft ground reaction force fluctuations, and that a lower SF should be associated to higher vertical and fore-aft ground reaction force fluctuations but to a lower extent than for DF (Bonnaerens et al., 2021). Moreover, we hypothesized that 2) both a lower DF and lower SF should be associated to higher peak forces (*F*_*z*,impact_, *F*_*z*,max_, *F*_brake,min_, *F*_prop,max_). Besides, higher DF runners demonstrated a more rearfoot strike pattern (Lussiana et al., 2019; Patoz et al., 2020) but should show lower vertical force than lower DF runners. Hence, we hypothesized that 3) the linearity of the force-length relationship should decrease with increasing DF, due to the higher chance to observe an impact peak when increasing DF, and that a higher DF should be associated to a lower $k_{leg}$ . Furthermore, we hypothesized that 4) a higher SF should correspond to a greater $k_{leg}$ and smaller leg compression, as previously observed (Morin et al., 2007; Coleman et al., 2012; Hobara et al., 2020).

## Materials and methods

### Participant characteristics

An existing database of 115 recreational runners (Patoz et al., 2021) including 87 males (age: 30 ± 8y, height: 180 ± 6 cm, leg length, measured from motion capture: 86 ± 4 cm, body mass: 70 ± 7 kg, weekly running distance: 38 ± 24 km, and running experience: 10 ± 8y) and 28 females (age: 30 ± 7 years, height: 169 ± 5 cm, leg length: 82 ± 4 cm, body mass: 61 ± 6 kg, weekly running distance: 22 ± 16 km, and running experience: 11 ± 8y) was used in this study. For study inclusion, participants were required to not have current or recent lower-extremity injury (≤1 month), to run at least once a week, and to have an estimated maximal aerobic speed ≥14 km/h (individual estimation). The study protocol was approved by the local Ethics Committee (CER-VD 2020–00334).

### Experimental procedure

After the participants provided written informed consent, retroreflective markers were positioned on the participants (described in Subsec. *Data Collection*) to record their running biomechanics. For each participant, a 1-s static trial was first recorded while he or she stood in a standard anatomical position on an instrumented treadmill (Arsalis T150–FMT-MED, Louvain-la-Neuve, Belgium) for calibration purposes. Then, a 7-min warm-up run was performed on the same treadmill (9–13 km/h). After a short break (<5min), three 1-min runs (9, 11, and 13 km/h) were performed in a randomized order (1-min recovery between each run). Three-dimensional (3D) kinematic and kinetic data were collected during the static trial and the last 30s of the running trials (83 ± 5 running steps), resulting in more than 20 steps being analyzed (Riazati et al., 2019). All participants were familiar with running on a treadmill, as it was part of their usual training program, and they wore their habitual running shoes (shoe mass: 256 ± 48 g and shoe heel-to-toe drop: 7 ± 3 mm).

### Data collection

Whole-body 3D kinematic data were collected at 200 Hz using motion capture (8 cameras) and Vicon Nexus software v2.9.3 (Vicon, Oxford, United Kingdom). The laboratory coordinate system was oriented such that the *x*-, *y*-, and *z*-axes denoted the mediolateral (pointing towards the right side of the body), posterior-anterior, and inferior-superior axes, respectively. Forty-three and 39 retroreflective markers of 12.5 mm diameter were used for the static and running trials, respectively. They were affixed to the skin and shoes of individuals on anatomical landmarks using double-sided tape following standard guidelines (Tranberg et al., 2011). Synchronized kinetic data (1000 Hz) were also collected using the force plate embedded into the treadmill.

The 3D marker and ground reaction force data (analog signal) were exported in the. c3d format and processed in Visual3D Professional software v6.01.12 (C-Motion Inc., Germantown, MD, United States). The 3D marker data were interpolated using a third-order polynomial least-square fit algorithm, allowing a maximum of 20 frames for gap filling, and were subsequently low-pass filtered at 20 Hz using a fourth-order Butterworth filter. The 3D ground reaction force signal was filtered using the same filter, and down sampled to 200 Hz to match the sampling frequency of the marker data.

A full-body biomechanical model with six degrees of freedom and 15 rigid segments was constructed from the marker set. The segments included the head, upper arms, lower arms, hands, thorax, pelvis, thighs, shanks, and feet. In Visual3D, the segments were treated as geometric objects, assigned inertial properties and COM locations based on their shape (Hanavan, 1964), and attributed relative masses based on standard regression equations (Dempster, 1955). The whole-body COM location was calculated from the parameters of all 15 segments (the whole-body COM was directly provided by Visual3D).

For all biomechanical measures, the values extracted from the 30-s data collection for each participant, including both right and left steps, were averaged for subsequent statistical analyses.

### Event detection

For each running trial, foot-strike, toe-off, and mid-stance events were identified with Visual3D. Foot-strike and toe-off were detected by applying a 20N threshold to the vertical ground reaction force (Smith et al., 2015). Mid-stance was placed at the instant where the fore-aft ground reaction force changed from negative to positive, which permitted to separate the stance phase in a braking and propulsive phase.

Temporal Variables $t_{c}$ , flight time ( $t_{f}$ ), and swing time ( $t_{s}$ ) were defined as the time from foot-strike to toe-off of the same foot, from toe-off of one foot to foot-strike of the contralateral foot, and from toe-off to foot-strike of the same foot, respectively.

DF was calculated as $DF=t_{c}/\left(t_{c}+t_{s}\right)$ , where $1/\left(t_{c}+t_{s}\right)$ represents the stride frequency (Minetti, 1998). SF was defined as the inverse of the sum of $t_{c}$ and $t_{f}$ , i.e., $SF=1/\left(t_{c}+t_{f}\right)$ . Furthermore, SF was normalized by $\sqrt{g/L_{0}}$ (Lieberman et al., 2015; van Oeveren et al., 2021), where *g* is the gravitational constant and *L*_*0*_ the leg length, calculated as the distance between hip and ankle joint center using the static (calibration) trial.

Braking ( $t_{brake}$ ) and propulsive ( $t_{prop}$ ) times were given as the time from foot-strike to mid-stance and mid-stance to toe-off of the same foot, respectively.

Compression ( $t_{comp}$ ) and decompression ( $t_{decomp}$ ) times were given as the time from foot-strike to the time where the vertical position of the whole-body COM is at its minimum, i.e., where the vertical ground reaction force is maximum, and from the time where the vertical position of the whole-body COM is at its minimum to toe-off, respectively.

### Ground reaction force variables

*F*_*z*,impact_ and *F*_*z*,max_ were obtained from the vertical ground reaction force signal (Luo et al., 2019). Noteworthy, an impact peak was not always observed, *F*_*z*,impact_ was quantified in 80% of the running trials. Besides, *F*_brake,min_ and *F*_prop,max_ were given by the minimum and maximum values of the fore-aft ground reaction force signal (Luo et al., 2019).

The instantaneous vertical loading rate (LR_*z*_) was calculated as the largest slope of the vertical ground reaction force signal between 20 and 80% of the first 15% of the stance phase (Willson et al., 2014). The 15% limit was chosen because an impact peak was not always identified and so that the loading rate of every runner was in the same relative temporal window (Willson et al., 2014). The braking (LR_brake_) and propulsive (LR_prop_) loading rates, because of their relation to running-related injuries (Daoud et al., 2012; Willson et al., 2014; Davis et al., 2016; Johnson et al., 2020), were calculated as the largest slopes of the fore-aft ground reaction force signal between foot-strike and the instant of *F*_brake,min_ and between mid-stance and the instant of *F*_prop,max_, respectively.

The braking (*I*_brake_) and propulsive (*I*_prop_) impulses were calculated as the integral of the fore-aft ground reaction force signal from foot-strike to mid-stance and from mid-stance to toe-off, respectively (Gottschall and Kram, 2005).

Force variables were all normalized by BW.

### Stiffness related variables

The spring-mass characteristics of the lower limb were assessed by computing the force-length relationship (Gill et al., 2020), i.e., the force vector projected along the leg as function of the leg compression/decompression during stance, and $k_{leg}$ (Liew et al., 2017), calculated using both the compression and decompression of the human body (Gill et al., 2020) and adapted from Liew et al. (2017). More explicitly, compressive (*k*_leg, comp_) and decompressive (*k*_leg, decomp_) leg stiffnesses were given by the maximum of the force vector projected along the leg (*F*_leg,max_) divided by the maximum leg compression (*∆L*_comp_) and decompression (*∆L*_decomp_) during stance, respectively. Following the definition of the spring-mass model, i.e., a massless spring attached to a point mass located at the whole-body COM (Blickhan, 1989), the leg length was represented by the magnitude of a 3D leg vector defined from the whole-body COM to the center of pressure of the foot. The center of pressure being subject to large fluctuations for low vertical force values, a 200N vertical threshold was used for foot-strike and toe-off events in this specific case (see supplementary materials). *∆L*_comp_ and *∆L*_decomp_ were given by the difference between the leg length at foot-strike and the minimum value of the leg length and by the difference between the leg length at toe-off and the minimum value of the leg length, respectively. A leg angle ( $\theta_{leg}$ ) was calculated as the angle between the leg vector and anterior-posterior axis, and evaluated at foot-strike ( $\theta_{leg,FS}$ ) and toe-off ( $\theta_{leg,TO}$ ) (Coleman et al., 2012).

*F*_leg,max_ was normalized by BW, *∆L*_comp_ and *∆L*_decomp_ were expressed in absolute and relative (as a percentage of participant’s height) units and similarly for *k*_leg, comp_ and *k*_leg, decomp_.

### Statistical analysis

All data are presented as the mean ± standard deviation. Pearson correlation coefficient (*r*) between DF and SF together with corresponding 95% confidence interval (lower, upper) were computed at the three running speeds separately. Correlations were considered *very high*, *high*, *moderate*, *low*, and *negligible* when absolute *r* values were between 0.90–1.00, 0.70–0.89, 0.50–0.69, 0.30–0.49, and 0.00–0.29, respectively (Hinkle et al., 2002). In this study, collinearity between DF and SF was prevented because *r* was smaller than 0.7 (Table 1) (Van Oeveren et al., 2019). The association of DF and SF on the vertical and fore-aft ground reaction force signals (along the entire stance phase) was examined using SPM and linear regression for each tested speed. Bonferroni correction was employed to consider that three running speeds were tested. To compare participants, the stance phase was normalized and therefore expressed in percentage. Besides, residual plots were inspected and no obvious deviations from homoscedasticity or normality were observed. Hence, the association of DF and SF (covariates) and running speed on temporal, ground reaction force, and stiffness related variables was evaluated using a linear mixed effects model fitted by restricted maximum likelihood. The within-subject nature was controlled for by including random effects for participants (individual differences in the intercept of the model). The fixed effects included running speed (categorical variable) and DF and SF (continuous variables). The linearity of the force-length relationship was quantified using the coefficient of determination ( $R^{2}$ ) during both leg compression ( $R_{comp}^{2}$ ) and decompression ( $R_{decomp}^{2}$ ). However, the calculation of $R^{2}$ was modified so that $R_{comp}^{2}$ and $R_{decomp}^{2}$ values were computed by comparing the compression and decompression force-length relationships to the perfectly elastic compression and decompression lines, i.e., linear relations obtained using slopes equal to *k*_leg, comp_ and *k*_leg, decomp_, respectively (Gill et al., 2020). In other words, $R^{2}$ evaluates how far the force-length relationship is from a linear model obtained using $k_{leg}$ . Statistical analysis was performed using spm1D (v0.4.6, https://spm1d.org) (Pataky, 2012), *Python* (v3.7.4, http://www.python.org), and Jamovi (v1.6.23, https://www.jamovi.org) with a level of significance set at *p* ≤ 0.05.

**TABLE 1:** Duty factor (DF) and step frequency (SF), as well as their Pearson’s correlation coefficient (*r*) together with their 95% confidence interval (lower, upper) and statistical significance (*P ≤* 0.05), indicated in bold, for three tested speeds.

Running speed (km/h)	DF (%)	SF (-)	*r*	*P*
9	37.7 ± 3.1	0.80 ± 0.04	0.32 (0.14, 0.47)	**<0.001**
11	34.7 ± 2.5	0.82 ± 0.04	0.33 (0.15, 0.48)	**<0.001**
13	32.6 ± 2.2	0.84 ± 0.04	0.32 (0.14, 0.47)	**<0.001**

Note: values are presented as mean ± standard deviation. SF was normalized by $\sqrt{g/L_{0}}$ , where *g* is the gravitational constant and *L*_*0*_ the leg length.

## Results

The increase of the running speed from 9 to 13 km/h was accompanied with a decrease of DF of 13.3 ± 3.8% and an increase of SF of 5.9 ± 3.1%. The correlation between DF and SF was *low* but significant at all tested speeds (*r* ≤ 0.32; *p* < 0.001; Table 1).

*t*_*c*_, *t*_brake_, *t*_prop_, *t*_comp_, and *t*_decomp_ significantly increased with increasing DF while *t*_*f*_ decreased (*p* < 0.001; Table 2). These six variables significantly decreased with increasing SF (*p* < 0.001; Table 2). Besides, *t*_*c*_ and *t*_decomp_ decreased with increasing speed while *t*_*f*_ increased with increasing speed (*p* ≤ 0.02; Table 2).

**TABLE 2:** Temporal variables for runners at endurance running speeds. Significant differences (*P* ≤ 0.05) identified by linear mixed effects modeling are indicated in bold. Note: values are presented as mean ± standard deviation. DF: duty factor, SF: step frequency, $t_{c}$ : contact time, $t_{brake}$ : brake time, $t_{prop}$ : propulsion time, $t_{comp}$ : compression time, $t_{decomp}$ : decompression time, and $t_{f}$ : flight time. SF covariate was normalized by $\sqrt{g/L_{0}}$ , where *g* is the gravitational constant and *L*_*0*_ the leg length. Up ( ↑ **)** and down ( ↓ ) arrows indicate positive and negative effects of the covariate, respectively. † and ‡ Significantly different from the value at 13 km/h.

Running speed (km/h)	*t*_*c*_ (ms)	*t*_brake_ (ms)	*t*_prop_ (ms)	*t*_comp_ (ms)	*t*_decomp_ (ms)	*t*_*f*_ (ms)
9	279 ± 24	139 ± 13	140 ± 14	113 ± 12	166 ± 18	92 ± 24^†^
11	250 ± 19	126 ± 11	124 ± 10	104 ± 11	146 ± 13	111 ± 20^‡^
13	228 ± 17	116 ± 10	112 ± 9	96 ± 10	132 ± 12	122 ± 18
Running speed effect (*P*)	**<0.001**	0.33	0.39	0.26	**0.02**	**<0.001**
DF covariate effect (*P*)	↑ **<0.001**	↑ **<0.001**	↑ **<0.001**	↑ **<0.001**	↑ **<0.001**	↓ **<0.001**
SF covariate effect (*P*)	↓ **<0.001**	↓ **<0.001**	↓ **<0.001**	↓ **<0.001**	↓ **<0.001**	↓ **<0.001**

**FIGURE 1:**
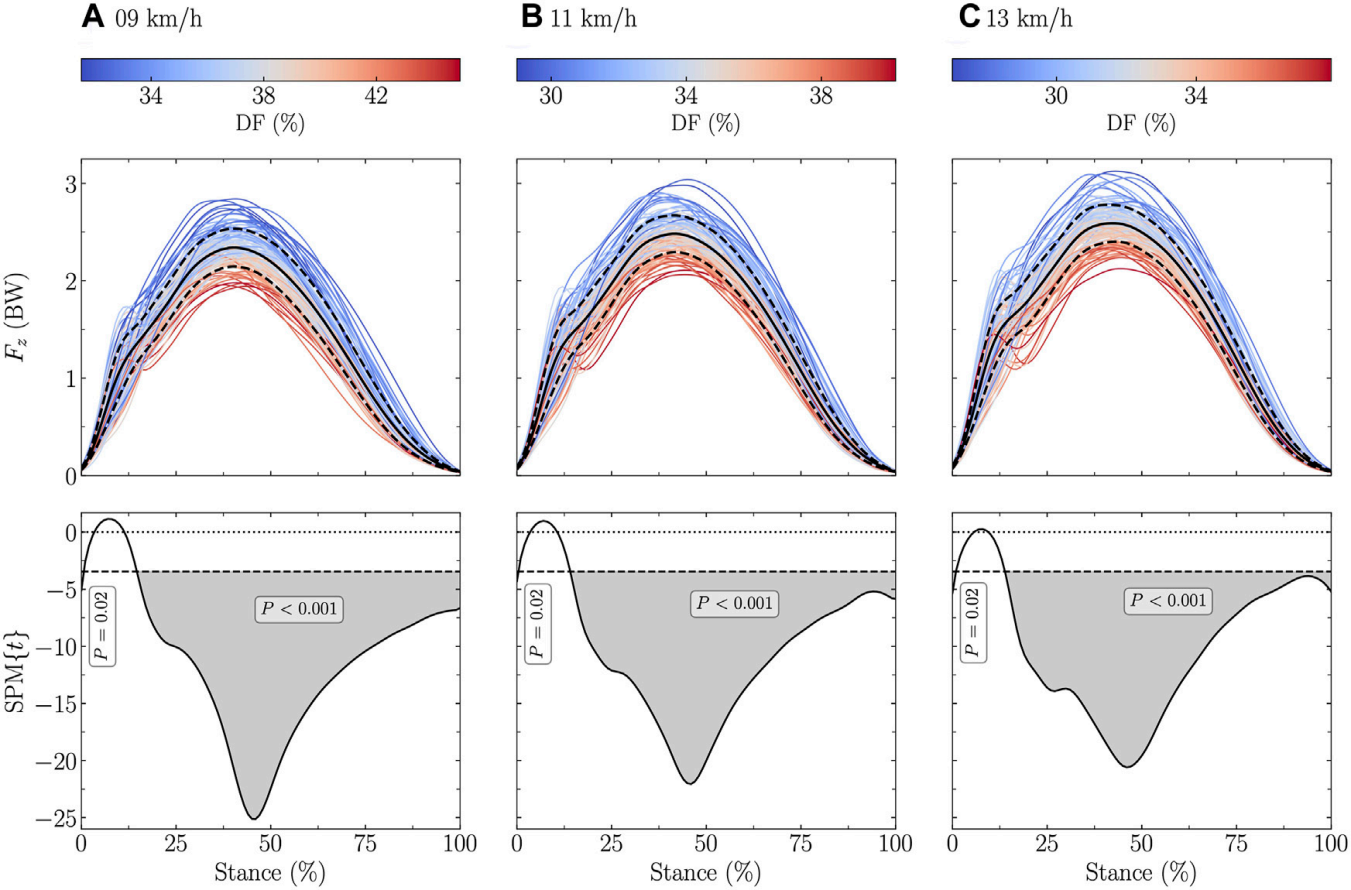
Statistical parametric mapping (SPM) analysis, i.e., t-statistics (SPM{t}), of the linear relationship between the vertical ground reaction force (F_z_) and the duty factor (DF) along the running stance phase at **(A)** 9 km/h, **(B)** 11 km/h, and **(C)** 13 km/h. In the upper panels, F_z_, expressed in body weight (BW), is depicted for each participant (the color depends on the DF value) and for the mean (black line) ± standard deviation (dashed black line) over all participants. In the lower panels, the black dashed horizontal lines represent the critical (parametric) threshold while the portion of the running stance phase which is statistically significant (*p* ≤ 0.017; Bonferroni correction was applied to take into the three tested speeds) is given by the gray shaded area.

**FIGURE 2:**
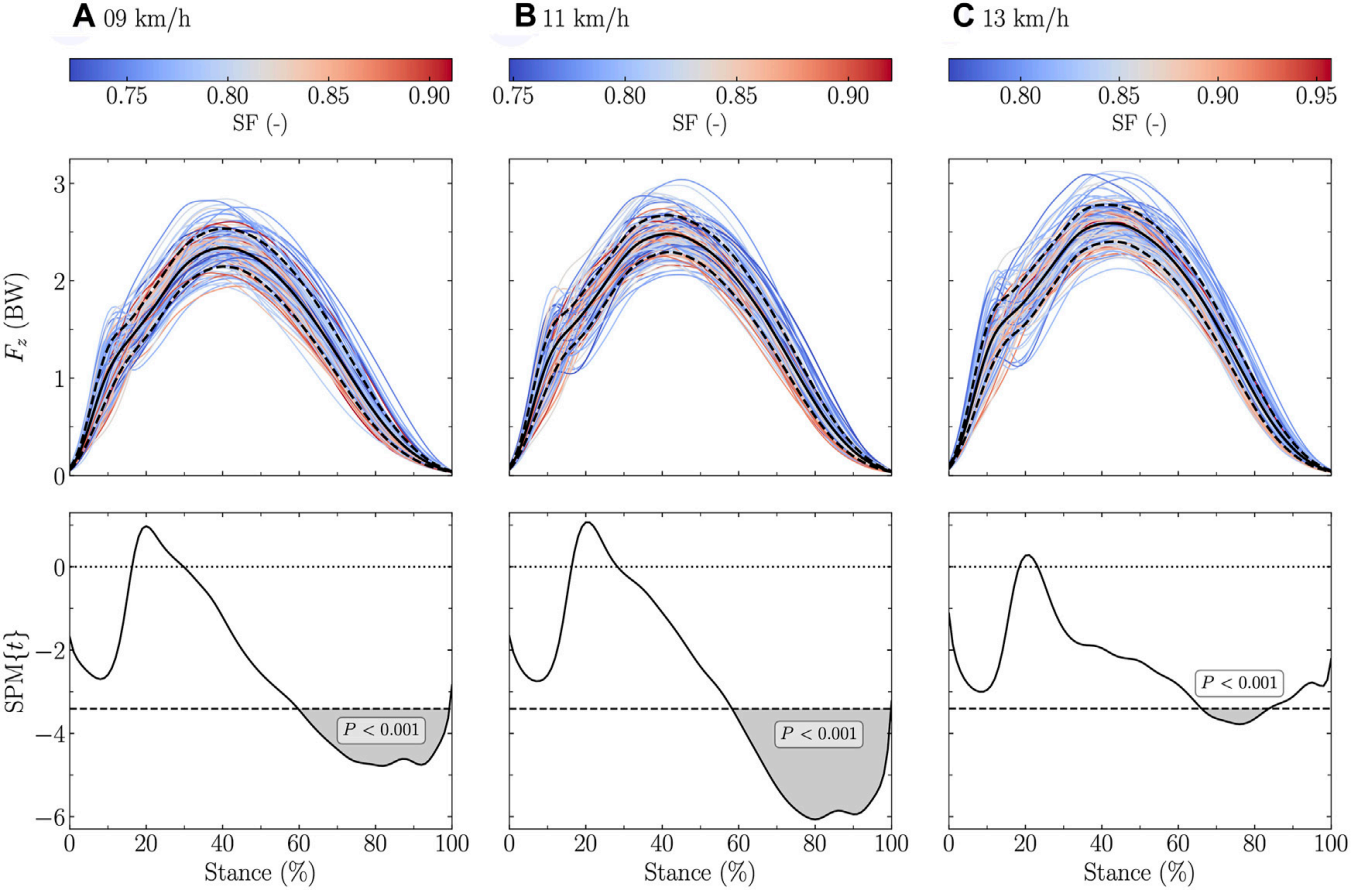
Statistical parametric mapping (SPM) analysis, i.e., t-statistics (SPM{t}), of the linear relationship between the vertical ground reaction force (F_z_) and the step frequency (SF) normalized by $\sqrt{g/L_{0}}$ , where g is the gravitational constant and L_0_ the leg length, along the running stance phase at **(A)** 9 km/h, **(B)** 11 km/h, and **(C)** 13 km/h. In the upper panels, F_z_, expressed in body weight (BW), is depicted for each participant (the color depends on the DF value) and for the mean (black line) ± standard deviation (dashed black line) over all participants. In the lower panels, the black dashed horizontal lines represent the critical (parametric) threshold while the portion of the running stance phase which is statistically significant (*p* ≤ 0.017; Bonferroni correction was applied to take into the three tested speeds) is given by the gray shaded area.

The vertical ground reaction force signal was significantly negatively related to DF at all tested speeds (stance range: 0 and 15–100% at 9 and 11 km/h, and 0 and 14–100% at 13 km/h; Figure 1). Similar findings were obtained for SF but to a lower extent (stance range: 60–99% at 9 km/h, 59–99% at 11 km/h, and 67–83% at 13 km/h; Figure 2).

**FIGURE 3:**
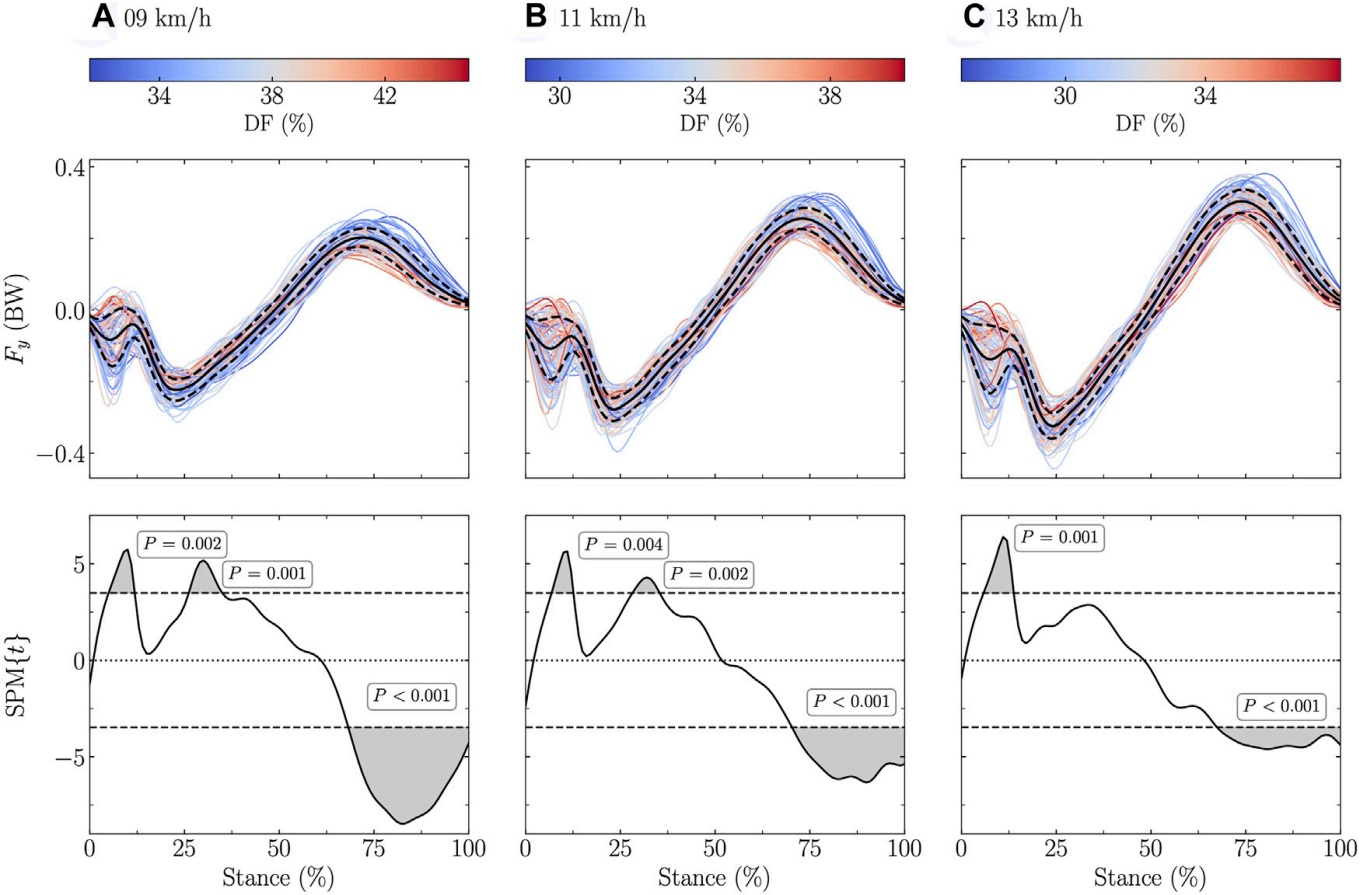
Statistical parametric mapping (SPM) analysis, i.e., t-statistics (SPM{t}), of the linear relationship between the fore-aft ground reaction force (F_y_) and the duty factor (DF) along the running stance phase at **(A)** 9 km/h, **(B)** 11 km/h, and **(C)** 13 km/h. In the upper panels, F_y_, expressed in body weight (BW), is depicted for each participant (the color depends on the DF value) and for the mean (black line) ± standard deviation (dashed black line) over all participants. In the lower panels, the black dashed horizontal lines represent the critical (parametric) threshold while the portion of the running stance phase which is statistically significant (*p* ≤ 0.017; Bonferroni correction was applied to take into the three tested speeds) is given by the gray shaded area.

**FIGURE 4:**
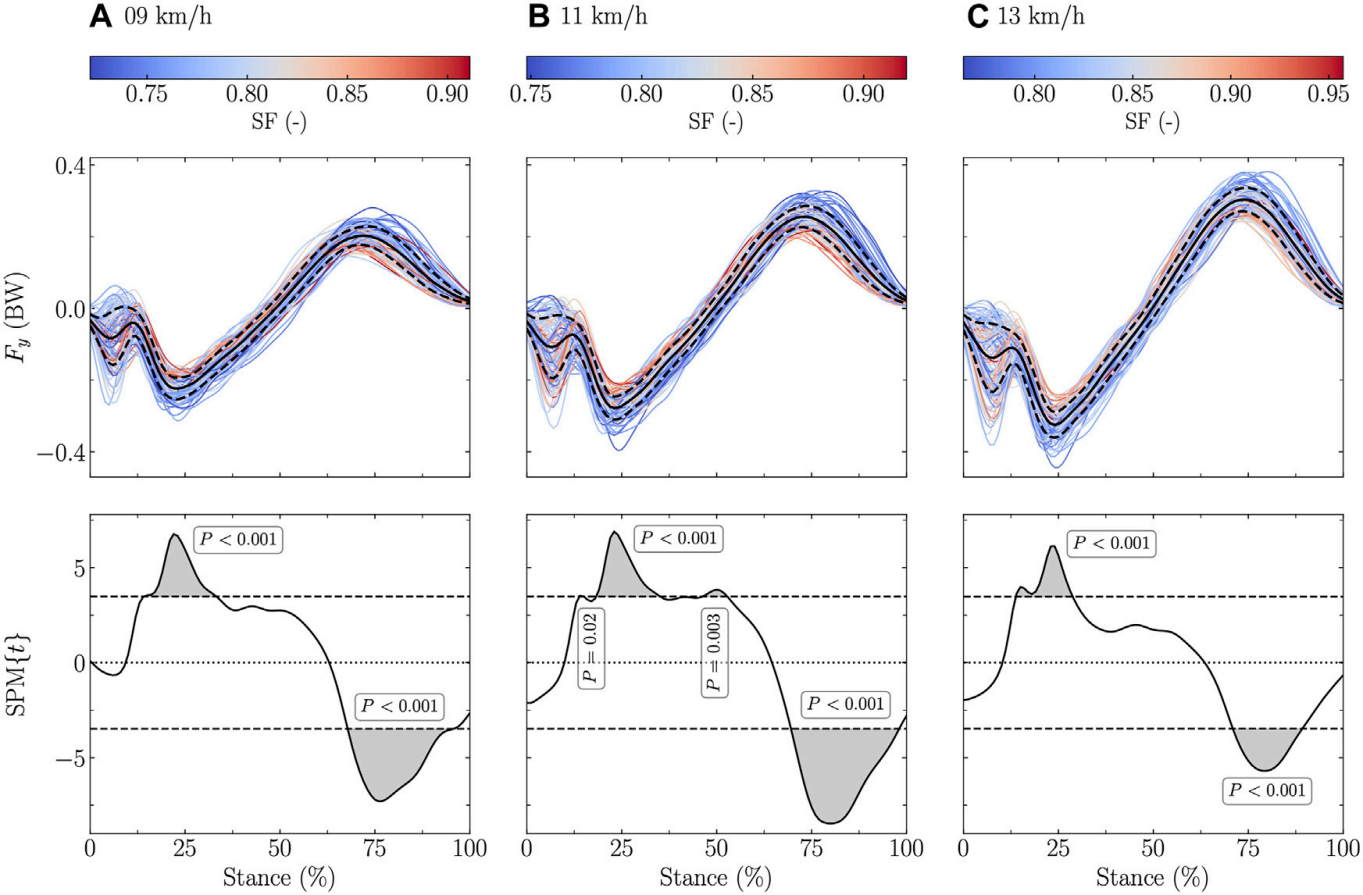
Statistical parametric mapping (SPM) analysis, i.e., t-statistics (SPM{t}), of the linear relationship between the fore-aft ground reaction force (F_y_) and the step frequency (SF) normalized by $\sqrt{g/L_{0}}$ , where g is the gravitational constant and L_0_ the leg length, along the running stance phase at **(A)** 9 km/h, **(B)** 11 km/h, and **(C)** 13 km/h. In the upper panels, F_y_, expressed in body weight (BW), is depicted for each participant (the color depends on the DF value) and for the mean (black line) ± standard deviation (dashed black line) over all participants. In the lower panels, the black dashed horizontal lines represent the critical (parametric) threshold while the portion of the running stance phase which is statistically significant (*p* ≤ 0.017; Bonferroni correction was applied to take into the three tested speeds) is given by the gray shaded area.

The fore-aft ground reaction force signal was significantly positively related to both DF and SF in the first 50% of the stance (negative fore-aft force) and negatively related to both DF and SF in the last 50% of the stance at all tested speeds (stance range for DF: 5–11, 27–34, and 69–100% at 9 km/h, 7–12, 29–35, and 71–100% at 11 km/h, and 6–13 and 68–100% at 13 km/h; Figure 3; stance range for SF: 15–33 and 68–95% at 9 km/h, 14, 19–35, 47–52, and 70–98% at 11 km/h, and 14–28 and 71–89% at 13 km/h; Figure 4).

*F*_*z*,max_, *F*_*z*,impact_, *F*_prop,max_, LR_prop_, and *I*_brake_ significantly decreased with increasing DF while *F*_brake,min_, LR_brake_, and *I*_prop_ significantly increased (*p* ≤ 0.01; Table 3). *F*_*z*,impact_, *F*_brake,min_, LR_prop_, and *I*_brake_ significantly increased with increasing SF while, *F*_prop,max_ and *I*_prop_ significantly decreased (*p* ≤ 0.02; Table 3). Considering absolute values, all the ground reaction force variables significantly increased with increasing speed (*p* ≤ 0.005; Table 3).

**TABLE 3:** Ground reaction force variables for runners at endurance running speeds. Significant differences (*P* ≤ 0.05) identified by linear mixed effects modeling are indicated in bold.

Running speed (km/h)	*F*_*z*,max_ (BW)	*F*_*z*,impact_ (BW)	*F*_brake,min_ (BW)	*F*_prop,max_ (BW)	LR_*z*_ (BW/s)	LR_brake_ (BW/s)	LR_prop_ (BW/s)	*I*_brake_ (BW ∙ s)	*I*_prop_ (BW ∙ s)
9	2.36 ± 0.19^a^ ^,^ ^b^	1.53 ± 0.28^a^ ^,^ ^b^	-0.24 ± 0.03^a^ ^,^ ^b^	0.21 ± 0.03^a^ ^,^ ^b^	49.0 ± 11.9^a^ ^,^ ^b^	-13.4 ± 2.7^a^ ^,^ ^b^	5.7 ± 0.8^a^ ^,^ ^b^	-0.016 ± 0.002^a^ ^,^ ^b^	0.017 ± 0.002^a^ ^,^ ^b^
11	2.50 ± 0.19	1.63 ± 0.30	-0.29 ± 0.03^b^	0.26 ± 0.03^b^	58.7 ± 13.4^b^	-16.2 ± 3.1^b^	7.5 ± 1.0^b^	-0.018 ± 0.002^‡^	0.019 ± 0.002^b^
13	2.62 ± 0.19	1.81 ± 0.32	-0.34 ± 0.03	0.31 ± 0.03	68.4 ± 15.2	-18.1 ± 3.4	9.7 ± 1.4	-0.019 ± 0.002	0.021 ± 0.002
Running speed effect (*P*)	**0.005**	**<0.001**	**<0.001**	**<0.001**	**<0.001**	**<0.001**	**<0.001**	**<0.001**	**<0.001**
DF covariate effect (*P*)	↓ **<0.001**	↓ **<0.001**	↑ **0.01**	↓ **<0.001**	0.34	↑ **<0.001**	↓ **<0.001**	↓ **0.004**	↑ **<0.001**
SF covariate effect (*P*)	0.33	↑ **<0.001**	↑ **<0.001**	↓ **<0.001**	0.11	0.17	↑ **0.02**	↑ **<0.001**	↓ **<0.001**

Note: values are presented as mean ± standard deviation. DF: duty factor, SF: step frequency, *F*_*z*,max_ and *F*_*z*,impact_: active and impact peaks, *F*_brake,min_: minimum braking force, *F*_prop,max_: maximum propulsive force, LR_*z*_: instantaneous vertical loading rate, LR_brake_: instantaneous braking loading rate, LR_prop_: instantaneous propulsive loading rate, and *I*_brake_ and *I*_prop_: braking and propulsive impulses. Ground reaction force variables were normalized by body weight (BW) and SF covariate was normalized by $\sqrt{g/L_{0}}$ , where *g* is the gravitational constant and *L*_*0*_ the leg length. Up ( ↑ **)** and down ( ↓ ) arrows indicate positive and negative effects of the covariate, respectively.

^a^ Significantly different from the value at 11 km/h.

^b^ Significantly different from the value at 13 km/h.

**FIGURE 5:**
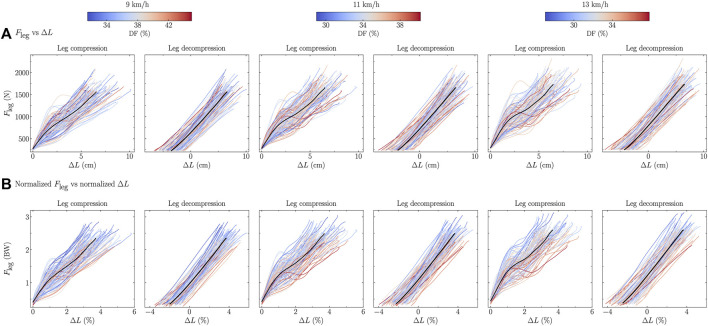
Force-length relationship, i.e., ground reaction force projected along the leg (*F*_leg_) as function of the leg compression/decompression, for each participant [the color depends on the duty factor (DF) value] and for the mean (black line) over all participants during the running stance phase, at three running speeds, and expressed using **(A)** SI units and **(B)** normalized units, i.e., body weight (BW) for *F*_leg_ and percentage of runners’ height for leg compression.

**FIGURE 6:**
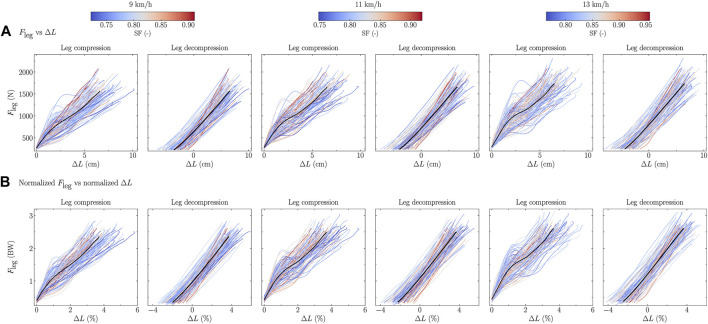
Force-length relationship, i.e., ground reaction force projected along the leg (*F*_leg_) as function of the leg compression/decompression, for each participant [the color depends on the step frequency (SF) value; SF was normalized by $\sqrt{g/L_{0}}$ , where *g* is the gravitational constant and *L*_*0*_ the leg length] and for the mean (black line) over all participants during the running stance phase, at three running speeds, and expressed using **(A)** SI units and **(B)** normalized units, i.e., body weight (BW) for *F*_leg_ and percentage of runners’ height for leg compression.

The force-length relationships of all participants, colored according to their DF and SF, are depicted in Figures 5, 6, respectively, for each tested speeds and separately for the compression and decompression phases. $R_{comp}^{2}$ significantly decreased with increasing DF or running speed, and increased with increasing SF (*p* ≤ 0.007; Table 4), while there was no change of $R_{decomp}^{2}$ with DF, SF, and speed.

**TABLE 4:** Linearity of the force-length relationship during leg compression ( $R_{comp}^{2}$ ) and decompression ( $R_{decomp}^{2}$ ). Significant differences (*p* ≤ 0.05) identified by linear mixed effects modeling are indicated in bold.

Running speed (km/h)	$R_{comp}^{2}$	$R_{decomp}^{2}$
9	0.95 ± 0.06^a^ ^,^ ^b^	0.99 ± 0.02
11	0.93 ± 0.08^b^	0.99 ± 0.01
13	0.90 ± 0.10	0.99 ± 0.01
Running speed effect (*P*)	**<0.001**	0.14
DF covariate effect (*P*)	↓ **<0.001**	0.06
SF covariate effect (*P*)	↑ **0.007**	0.85

Note: values are presented as mean ± standard deviation. DF: duty factor, SF: step frequency, SF covariate was normalized by $\sqrt{g/L_{0}}$ , where *g* is the gravitational constant and *L*_*0*_ the leg length. Up ( ↑ ) and down ( ↓ ) arrows indicate negative effects of the covariate.

^a^ Significantly different from the value at 11 km/h.

^b^ Significantly different from the value at 13 km/h.

*F*_*z*,max_, *∆L*_comp_, *k*_leg, comp_, and *k*_leg, decomp_ significantly decreased with increasing DF while *∆L*_decomp_ and $\theta_{leg,TO}$ significantly increased (*p* ≤ 0.03; Table 5). *∆L*_comp_, *∆L*_decomp_, and $\theta_{leg,TO}$ significantly decreased with increasing SF while *k*_leg, comp_, and *k*_leg, decomp_ significantly increased (*p* < 0.001; Table 5). *F*_leg,max_, *∆L*_decomp_, *k*_leg, decomp_, ${\left|\theta \right.}_{leg,FS}$ |, and $\theta_{leg,TO}$ significantly increased with increasing speed (*p* ≤ 0.005; Table 5).

**TABLE 5:** Stiffness variables for runners at endurance running speeds. Significant differences (*P* ≤ 0.05) identified by linear mixed effects modeling are indicated in bold.

Running speed (km/h)	*F*_leg,max_ (BW)	*∆L*_comp_ (cm)	*∆L*_decomp_ (cm)	*∆L*_comp_ (%)	*∆L*_decomp_ (%)	*k*_leg, comp_ (kN/m)	*k*_leg, decomp_ (kN/m)	*k*_leg, comp_ (BW/%)	*k*_leg, decomp_ (BW/%)	$\theta_{leg,FS}$ (deg)	$\theta_{leg,TO}$ (deg)
9	2.36 ± 0.19^a^ ^,^ ^b^	6.5 ± 1.1	10.0 ± 1.0^a^ ^,^ ^b^	3.7 ± 0.6	5.7 ± 0.6^a^ ^,^ ^b^	24.6 ± 4.5	15.8 ± 2.4^a^ ^,^ ^b^	0.66 ± 0.11	0.42 ± 0.06^a^ ^,^ ^b^	-8.1 ± 2.6^a^ ^,^ ^b^	13.4 ± 1.6^a^ ^,^ ^b^
11	2.50 ± 0.19	6.5 ± 1.1	10.4 ± 1.1^b^	3.7 ± 0.6	5.9 ± 0.6^b^	26.3 ± 5.0	16.2 ± 2.4^b^	0.70 ± 0.13	0.43 ± 0.06^b^	-9.2 ± 2.5^b^	15.0 ± 1.5^b^
13	2.62 ± 0.19	6.4 ± 1.1	10.7 ± 1.1	3.6 ± 0.6	6.1 ± 0.6	27.8 ± 5.3	16.3 ± 2.3	0.74 ± 0.13	0.44 ± 0.06	-10.2 ± 2.4	16.5 ± 1.5
Running speed effect (*P*)	**0.005**	0.38	**<0.001**	0.39	**<0.001**	0.30	**<0.001**	0.13	**<0.001**	**<0.001**	**<0.001**
DF covariate effect (*P*)	↓ **<0.001**	↓ **0.02**	↑ **0.03**	↓ **0.01**	↑ **0.01**	↓ **<0.001**	↓ **<0.001**	↓ **<0.001**	↓ **<0.001**	0.59	↑ **<0.001**
SF covariate effect (*P*)	0.36	↓ **<0.001**	↓ **<0.001**	↓ **<0.001**	↓ **<0.001**	↑ **<0.001**	↑ **<0.001**	↑ **<0.001**	↑ **<0.001**	0.83	↓ **<0.001**

Note: values are presented as mean ± standard deviation. DF: duty factor, SF: step frequency, *F*_leg,max_: maximum of the force vector projected along the leg, *∆L*_comp_ and *∆L*_decomp_: maximum leg compression and decompression during stance, *k*_leg, comp_ and *k*_leg, decomp_: compressive and decompressive leg stiffnesses, $\theta_{leg,FS}$ and $\theta_{leg,TO}$ : leg angle at foot-strike and toe-off. *F*_leg,max_ was normalized by body weight (BW). *∆L*_comp_ and *∆L*_decomp_ were expressed in absolute and relative (as a percentage of participant’s height) units and similarly for *k*_leg, comp_ and *k*_leg, decomp_. SF covariate was normalized by $\sqrt{g/L_{0}}$ , where *g* is the gravitational constant and *L*_*0*_ the leg length. Up ( ↑ **)** and down ( ↓ ) arrows indicate positive and negative effects of the covariate, respectively.

^a^ Significantly different from the value at 11 km/h.

^b^ Significantly different from the value at 13 km/h.

## Discussion

According to the first hypothesis, lower DF and lower SF were associated to higher vertical and fore-aft ground reaction force fluctuations, but SF to a lower extent than DF. Besides, according to the second hypothesis, larger *F*_*z*,max_, *F*_*z*,impact_, |*F*_brake,min_|, and *F*_prop,max_ were reported for lower DF values as well as larger |*F*_brake,min_|, and *F*_prop,max_ for lower SF values. However, there was no association between SF and *F*_*z*,max_ and a larger *F*_*z*,impact_ was reported for higher SF values, which partly refuted the second hypothesis. The linearity of the force-length relationship during the leg compression decreased with increasing DF but did not change during the leg decompression, partly refuting the third hypothesis. According to the fourth hypothesis, a higher SF was associated to a larger $k_{leg}$ and a smaller leg compression.

DF was previously analytically shown to be inversely proportional to the maximum of an approximated, based on a sine-wave model (Beck et al., 2020), vertical ground reaction force signal (Morin et al., 2005). This previous knowledge is further expanded by the present results which showed that *F*_*z*,max_ is significantly negatively related to DF (Table 3), and corroborates previous findings which showed that DF was negatively correlated to *F*_*z*,max_ (Bonnaerens et al., 2021). This suggests that DF should be inversely related to *F*_*z*,max_ without using a sine-wave model to approximate the vertical ground reaction force. Moreover, the present study extends to the fact that a lower DF results in a larger vertical ground reaction force during most of the stance (∼15–100%; Figure 1) but after the 15% temporal window representative of the “impact” phase (Willson et al., 2014). Therefore, the SPM analysis additionally revealed that the association between DF and the vertical ground reaction force signal is not only given at *F*_*z*,max_ but through almost the entire stance (after the impact phase; ≥15%). This result suggests that the shape of the vertical ground reaction force during the impact phase is not affected by the DF. This result might be attributed to the fact that the vertical ground reaction force signal is given by the force contributions of two discrete body mass components, i.e., a distal mass composed of the foot and shank and the remaining mass (Clark et al., 2017; Udofa et al., 2019). Hence, the impact phase, represented by the distal mass in this model, might not be affected by the DF. This study also showed that for the runners having a visible *F*_*z*,impact_, this *F*_*z*,impact_ was significantly larger for lower than higher DF values (Table 3). This discrepancy could be explained by fact that the impact peak might be happening at a different instant of the running stance phase (within the first 15%) depending on individuals.

Similarly, a lower SF resulted in a larger vertical ground reaction force, but only at the end of the stance (∼65–95%; Figure 2). This was likely related to the longer step length for running at the same speed. In fact, it has previously been shown that a larger vertical ground reaction force (i.e., support force) produces a larger step length (Weyand et al., 2000; Dorn et al., 2012). Our SPM analysis demonstrated that this larger support force was located only at the end of the stance. Indeed, *F*_*z*,max_ was not related to SF (Table 3). Non-etheless, the reason why this larger support force was located at the end of the stance could not readily be explained. Besides, *F*_*z*,impact_ significantly increased with increasing SF (Table 3). This result contradicts previous findings which observed a decrease of the impact peak with increasing SF (Lieberman et al., 2015). However, these findings were obtained when asking individuals to voluntarily increase their SF. Hence, this could lead to a different running pattern than the spontaneous running pattern of runners with a naturally high SF.

The present study reported no association of DF and SF on LR_*z*_ (Table 3). This could partly follow from the fact that the SPM analysis did not report any significant association between DF and SF and the vertical ground reaction force during the impact phase (the first 15% of the stance). This result corroborates the absence of correlation between LR_*z*_ and both DF and SF at slow running speeds, as reported by Bonnaerens et al. (2021). However, assuming that DF is partly related to foot-strike pattern, i.e., the higher the DF, the more likely that this runner is a rearfoot striker (Lussiana et al., 2019; Patoz et al., 2020), this result contradicts the result of a meta-analysis which reported higher LR_*z*_ for rearfoot than non-rearfoot strikers (Almeida et al., 2015).

The fore-aft ground reaction force signal was positively related to DF around ∼5–10% of the stance and to both DF and SF around ∼25–35% (positively) and ∼70–90% (negatively; Figures 3, 4). The positive association of DF on the fore-aft ground reaction force signal reported by the SPM analysis around ∼5–10% of the stance can be explained by the foot-strike pattern. Indeed, fore-foot strikers were shown to have a negative spike on the fore-aft ground reaction force signal around ∼5–10% of the stance (Nordin et al., 2017) and DF was related to the footstrike pattern (Lussiana et al., 2019; Patoz et al., 2020). However, the association of DF on the fore-aft force signal around ∼5–10% of the stance was not accompanied by an association of DF on the vertical force signal at the same percentage of the stance. This suggests that the effect of DF during the impact phase was more important in the fore-aft than vertical force signal. The other two significant regions are around the braking and propulsive peaks (*F*_brake,min_ and *F*_prop,max_), which were also significantly related to DF and SF (Table 3). These results partly corroborate previous observations, which showed that the peak braking force was correlated to DF but not to SF (Bonnaerens et al., 2021). Moreover, they confirm that larger ground reaction forces during propulsion are needed to lift and accelerate the body during stance to generate longer step lengths (Schache et al., 2014). As previously suggested (van Oeveren et al., 2021), combining vertical and horizontal ground reaction forces into a single vector could be useful to properly characterize their orientations and actions and carefully describe the relationship of this single vector with DF and SF, especially at the end of the stance.

The linearity of the force-length relationship was higher for lower DF and SF than for higher DF and SF runners during the leg compression but there was no difference during the leg decompression (Table 4). This means that higher DF and SF values were associated to more variations of the instantaneous compressive stiffness, i.e., the slope for each pair of point during the leg compression. However, the decompressive stiffness during the leg decompression was independent of DF and SF (Table 4). This result corroborates the choice made by several authors to use the decompression phase instead of the compression one to calculate the vertical stiffness (Cavagna et al., 1988; Schepens et al., 1998). Deviation from linearity of the force-length relationship among individuals was also reported by Gill et al. (2020). Indeed, these authors reported that the linearity of the force-length curve was foot-strike index (foot-strike pattern) dependent and that this curve should be investigated before using the spring-mass model. Furthermore, these authors suggested that for $R^{2}<0.95$ , it may be more appropriate to segment the stance phase and to individually investigate the different subphases. Hence, the deviation from linearity observed herein during the leg compression for higher than lower DF and SF runners suggest that the stiffness should be split into several phases during the leg compression and thus invalidate the usage of *k*_leg, comp_ for these runners. However, the linearity observed during leg decompression for all participants suggest that *k*_leg, decomp_ could be used.

This study reported that *k*_leg, decomp_ and *F*_leg,max_ significantly increased with decreasing DF while *∆L*_decomp_ decreased (Table 5). Hence, the elastic energy (*E*_el_), which could be calculated as $E_{el}=\frac{F_{leg,\max}^{2}k_{leg,decomp}}{2}$ using the definition of the spring-mass model, increased with decreasing DF. Furthermore, the compression was more vertical and $t_{c}$ was shorter for lower than higher DF runners. High DF runners could be characterized by a slow stretch-shortening cycle (runners with $t_{c}$ longer than 250 m) while low DF runners by a fast one (Vogt and Hoppeler, 2014). These results, together with the higher linearity of the force-length curve during the compression phase observed for lower than higher DF runners suggest that a lower DF runner better optimizes the spring-mass model than a higher DF runner. On the contrary, $\theta_{leg,TO}$ significantly increased with increasing DF (Table 5). These results suggest that the higher the DF, the higher the promotion of forward propulsion of the body. This compensates for the lower utilization of the spring-mass model of higher than lower DF runners and corroborates previous findings (Lussiana et al., 2019; Patoz et al., 2020). These findings bring further evidence and reinforce previous statements that low DF runners rely more on the optimization of the spring-mass model whereas high DF runners promotes forward propulsion (pulley system) (Lussiana et al., 2019; Patoz et al., 2020).

This study further revealed that the higher the SF, the larger *k*_leg, decomp_ and the smaller *∆L*_decomp_ (Table 5), which corroborates previous findings (Coleman et al., 2012; Hobara et al., 2020). Moreover, $\theta_{leg,TO}$ significantly decreased with increasing SF (Table 5). These results suggest that higher SF runners better optimize the spring-mass model than lower SF runners, confirming that SF seems to be an indirect factor influencing *k*_leg_ through its effect on $t_{c}$ (Morin et al., 2007).

Most of the variables studied herein reported an opposite association of DF and SF covariates (Tables 3-5). In other words, for most of the variables, if DF had a positive association on a given variable, then SF had a negative association on the same variable, and *vice versa*. This observation sounds counter-intuitive because SF is analytically associated to DF, i.e., $DF={0.5\;t}_{c}\;SF$ . However, though significant, correlations between DF and SF were *low* at all tested speeds (Table 1). Hence, the direct association of SF covariate on a given variable is more important than the indirect association caused by the relationship between SF and DF. Besides, the *low* correlations between SF and DF tend to reduce the direct association of a covariate on a given variable. Noteworthy, correlations between DF and $t_{c}$ were *high* (*r* ≥ 0.78) and significant (*p* < 0.001) and correlations between DF and $t_{f}$ were *very high* (*r* ≥ 0.95) and significant (*p* < 0.001). Hence, these results corroborate that DF and SF can be viewed as two variables that complement each other and that should be used together to describe the full spectrum of running patterns (van Oeveren et al., 2021).

A few limitations to the present study exist. Few findings of this study were obtained using the spring-mass model, which include many assumptions and limitations (Blickhan, 1989; McMahon and Cheng, 1990; Farley and González, 1996) that may restrict our conclusion on the underlying mechanisms. However, due to the methodological challenges associated to *in vivo* measurements under dynamic conditions to understand the role of muscle-tendon unit during running, the use of spring-loaded inverted pendulum model seems rational and relevant. In addition, the running speeds were limited to endurance speeds representative of the running speeds employed by recreational runners during endurance running training (Selinger et al., 2022) and experimental trials were performed on a treadmill. Similar results might also be obtained using overground running trials because spatiotemporal parameters between motorized treadmill and overground running are largely comparable (Van Hooren et al., 2020). However, it was also concluded that participants behaved differently when attempting to achieve faster speeds overground than on a treadmill (Bailey et al., 2017). Therefore, further studies should investigate the association of DF and SF on running kinetic using additional conditions, i.e., faster speeds, positive and negative slopes, and different types of ground.

To conclude, this study revealed that the lower the DF and the higher the SF, the more the runner relies on the optimization of the spring-mass model, whereas the higher the DF and the lower the SF, the more the runner promotes forward propulsion.

## Data Availability

The raw data supporting the conclusions of this article will be made available by the authors, without undue reservation.
