# Erratum: Using statistical parametric mapping to assess the association of duty factor and step frequency on running kinetic

**DOI:** 10.3389/fphys.2023.1171196

**Published:** 2023-03-02

**Authors:** Frontiers Production Office

**Affiliations:** Frontiers Media SA, Lausanne, Switzerland

**Keywords:** biomechanics, running pattern, spring-mass model, leg stiffness, ground reaction force

An omission to the **Funding** section of the original article was made in error. The following sentence has been added: “Open access funding was provided by the University of Lausanne.”

The original version of this article has been updated.

