# Supplementary material for: Using statistical parametric mapping to assess the association of duty factor and step frequency on running kinetic
Source: Front Physiol. 2022 Dec 5;13:1044363. doi: 10.3389/fphys.2022.1044363 (PMC9760857; doi:10.3389/fphys.2022.1044363)
Supplement: Supplementary file 1 [file Image1.pdf]

Supplementary Materials:

Using statistical parametric mapping to assess the association of duty factor and step frequency on running kinetic

S1 Trajectory of the Center of Pressure during the Stance Running Phase

Figure S1 shows the trajectory of the center of pressure for one representative right foot contact at 11km/h. This figure depicts that the center of pressure calculated with low vertical ground reaction force values, i.e., below 200N, are not reliable. This can be explained because the vertical ground reaction force is present in the denominator of the equations required to calculate the anterior-posterior and mediolateral trajectories of the center of pressure and therefore cannot be too low (reference: http://www.kwon3d.com/theory/grf/cop.html).

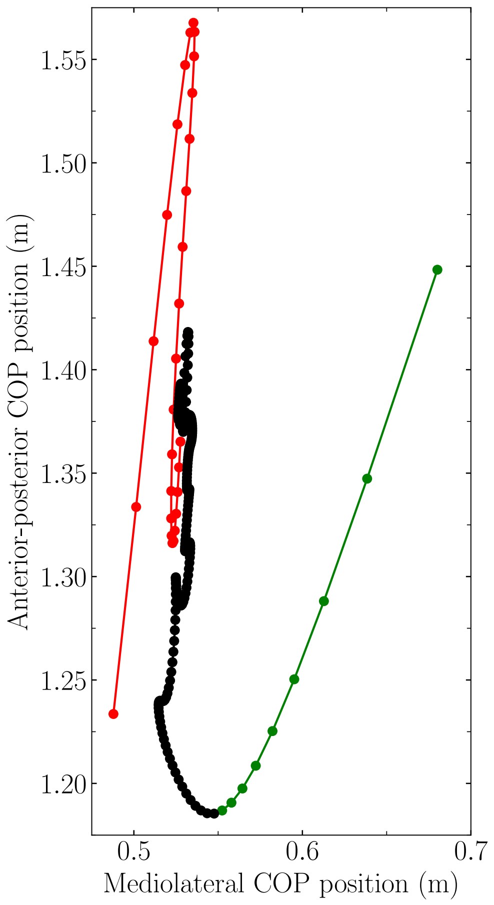

Figure S1. Example of the trajectory of the center of pressure (COP) for one representative right foot contact at 11km/h. The green and red colors show the trajectory of the COP at the initial and final instant of the stance running phase, respectively, i.e., when the vertical ground reaction force was below 200N. The vertical axis is the anterior-posterior COP position while the horizontal axis is the mediolateral COP position.
